# Draft Genome Sequences of Two *Salmonella* Strains from the SARA Collection, SARA64 (Muenchen) and SARA33 (Heidelberg), Provide Insight into Their Antibiotic Resistance

**DOI:** 10.1128/genomeA.00806-13

**Published:** 2013-10-03

**Authors:** Brenda S. Kroft, Eric W. Brown, Jianghong Meng, Narjol Gonzalez-Escalona

**Affiliations:** Division of Microbiology, Center for Food Safety and Applied Nutrition, Food and Drug Administration, College Park, Maryland, USAa; Department of Nutrition & Food Science, University of Maryland, College Park, Maryland, USAb

## Abstract

The *Salmonella enterica* strains that are representatives of the *S. enterica* serovar Typhimurium complex in reference collection A (SARA) are closely related but exhibit differences in antibiotic resistance, which could have public health consequences. To better understand the mechanisms behind these resistances, we sequenced the genomes of two multidrug-resistant strains: SARA64 (Muenchen) and SARA33 (Heidelberg).

## GENOME ANNOUNCEMENT

*Salmonella enterica* is one of the most important bacterial enteric pathogens and has been implicated in food-borne illnesses worldwide (1). Emergence of widespread multidrug resistance (MDR) among these strains could have a significant impact on public health (2, 3). Exploration of a well-characterized salmonella reference collection (4, 5), consisting of 72 representatives of the *S. enterica* serovar Typhimurium complex, revealed inherent resistance to antibiotics among some SARA strains (B. S. Kroft, unpublished data).

A phenotypic analysis of antimicrobial susceptibility on a subset of 63 strains of the SARA collection revealed that only 20 of those strains showed resistance to one or more antibiotics (Kroft, unpublished). Two of these strains, SARA64 and SARA33, exhibited resistance to ampicillin, chloramphenicol, tetracycline, streptomycin, sulfisoxazole, and kanamycin. SARA33 also showed resistance to gentamicin. Both strains were positive for the integrase found in integrons class I (*intI1*) (6, 7); however, analysis by PCR showed that SARA33 lacked the first gene in the integron cassette (8). In order to determine which genes and/or integrons are responsible for resistance, we sequenced the genomes of both SARA64 and SARA33.

DNA from each strain was isolated from overnight cultures with a DNeasy Blood and Tissue kit (Qiagen, Valencia, CA). The genomes were sequenced using an Ion Torrent (PGM) sequencing system with the 200-bp reads chemistry (Life Technologies, Carlsbad, CA) at 30 to 40× coverage, using an Ion PGM 200 sequencing kit, according to the manufacturer’s instructions. Genomic sequence contigs for each strain were *de novo* assembled using CLC Genomics Workbench version 5.5.1 (CLC bio, Germantown, MD). The G+C mol% values of SARA64 and SARA33 were 52.0 and 52.1%, respectively, which are similar to the reported GC content for other *Salmonella* strains (9). Strain SARA64 has 131 contigs, ranging from 501 to 287,053 bp, with a total size of 4,819,637 bp. SARA33 has 176 contigs, ranging from 513 to 168,116 bp, with a total size of 4,975,340 bp. A reference mapping approach using CLC Genomics Workbench showed that SARA33 carried a similar plasmid to pSL476_91 (pSL476_91) and SARA64 carried a highly similar plasmid (99.9% identity) to TY474p3 (CP002490.1).

These draft genome sequences were annotated using the NCBI Prokaryotic Genomes Automatic Annotation Pipeline (PGAAP) (http://www.ncbi.nlm.nih.gov/genomes/static/Pipeline.html) (10). Identity of the strains was confirmed by *in silico* multilocus sequence typing (MLST) (http://cge.cbs.dtu.dk/services/) (11) using the *Salmonella* MLST database (5); strain SARA33 was ST-1615 and SARA64 was ST-82 as reported previously (5). Antibiotic resistance genes were detected by *in silico* screening (12). SARA64 carried resistance genes for aminoglycosides [*aac*(*6′*)*-laa*, *aph*(*3′*)*-la*, *strA*, *strB*, and *aadA1*], sulfonamides (*sul1* and *sul2*), beta-lactams (*blaOXA*), tetracycline (*tetB*), and phenicol (*catA1*), which explain its MDR phenotype. Some of these resistance genes were on a genomic island similar to GI-DT12 in *Salmonella enterica* serovar Typhimurium T000240 (13), which contains an identical integron cassette (*intI1-blaOXA-aadA1-qacEΔ1-sul1*). The genes *strA*, *strB*, and *sul2* were located on a plasmid.

In contrast, SARA33 carried resistance genes for aminoglycosides [*aac*(*6′*)*-ly*, *aadA5*, *aadB*, *aa*(*6′*)*-33*, and *aadA1*], sulfonamides (*sul1* and *sul2*), beta-lactams (*blaOXA-2* and *blaTEM*), and tetracycline (*tetD*). A novel integron cassette was identified that contained one hypothetical protein (hp) with unknown function, followed by *aadA1* and *aa*(*6′*)*-33* genes [*intI1-hp-aac*(*6′*)*-33-aadA1*].

### Nucleotide sequence accession numbers.

The draft genome sequences of these two *Salmonella enterica* strains are now available in GenBank under accession numbers AUQD00000000 for strain SARA33 (2213-Heidelberg) and AUQE00000000 for strain SARA64 (2244-Muenchen).
